# Biomodulatory Treatment Regimen, MEPED, Rescues Relapsed and Refractory Classic Hodgkin’s Disease

**DOI:** 10.3389/fphar.2021.599561

**Published:** 2021-06-18

**Authors:** Florian Lüke, Dennis C. Harrer, Karin Menhart, Daniel Wolff, Ernst Holler, Dirk Hellwig, Wolfgang Herr, Matthias Grube, Martin Vogelhuber, Albrecht Reichle, Daniel Heudobler

**Affiliations:** ^1^ Department of Internal Medicine III, Hematology and Oncology, University Hospital of Regensburg, Regensburg, Germany; ^2^ Department of Nuclear Medicine, University Hospital of Regensburg, Regensburg, Germany

**Keywords:** metronomic low dose chemotherapy, everolimus, piogliatazone, etoricoxib, anakoinosis, r/r Hodkin's disease

## Abstract

**Introduction:** Current combined intensive chemotherapy and radiation regimens yield excellent survival rates in advanced classic Hodgkin’s lymphoma (cHL). However, acute toxicity in elderly, comorbid patients can be challenging and long-term survival in refractory patients remains poor.

**Patients and Methods:** We report on six patients with r/r HL, three patients with long-term follow-up, three newly treated, after biomodulatory therapy. All patients received MEPED (treosulfan 250 mg p.o. daily, everolimus 15 mg p.o. daily to achieve serum trough levels of 15 ng/ml, pioglitazone 45 mg p.o. daily, etoricoxib 60 mg p.o. daily and dexamethasone 0.5 mg p.o. daily). Patients had either received every at that time approved systemic treatment or were ineligible for standard treatment, including immune checkpoint inhibition (ICPi) due to prior demyelinating autoimmune polyneuropathy, myasthenia gravis and previous allogeneic hematopoietic-stem-cell transplant (alloHSCT). Medication was administered continuously from day 1. One patient with relapse after alloHSCT received trofosfamide 50 mg daily instead of treosulfan to avoid risk of increased myelotoxicity. The patients were treated in individual healing attempts outside a clinical trial after institutional review board approval. ^18^F-fluoro-2-deoxy-d-glucose positron emission tomography combined with computed tomography scan (FDG-PET/CT) was performed to monitor treatment and follow-up.

**Results:** In the three newly treated patients, CT scans showed partial remissions after 2–5 months on MEPED treatment. Two patients had achieved PET Deauville score 2 and 3, while the third remained positive at Deauville score 5. One patient achieving PR became eligible for alloHSCT, while the other two patients continued treatment with MEPED. All patients eventually achieved continuous complete remission (cCR), one after consecutive alloHSCT, one after discontinuing MEPED consolidation for >1 year and one on on-going MEPED consolidation, respectively. Only one patient experienced Grade 3 toxicity (bacterial pneumonia) requiring temporary discontinuation of MEPED for 10 days. All three previously published patients received allo HSCT for consolidation and have achieved cCR.

**Conclusions:** MEPED is well tolerated with low toxicity and highly efficacious in relapsed/refractory cHL, including severely comorbid patients. Due to its immunomodulatory components, MEPED might also have a synergistic potential when combined with ICPi but requires further evaluation within a clinical trial.

## Introduction

Classical Hodgkin’s lymphoma (cHL) usually occurs in adolescents and younger adults with the age peak being around 32 years. An annual incidence of 2–3/100,000 per year makes it the most frequent lymphoma in young adults in the Western World (Mottok and Steidl, 2018). In people aged >60 years there is also an increase in incidence, making this disease a disease of the elderly, especially in light of a rising life expectancy in the Western World.

Modern anthracycline-based treatment regimens with or without radiation currently cure beyond 80% of patients, especially in low-risk situations and in early stages of disease. Patients with high risk/advanced stages on the other hand are only cured in 70% of cases (Rathore und Kadin 2010; Dalal et al., 2020). While chemotherapy can also be used in patients >60 years of age with good treatment results, some of the elderly patients do not tolerate ABVD, the least aggressive and commonly used chemotherapeutic treatment approach for this age group.

High dose-chemotherapy with autologous stem cell transplantation (auto-HSCT) rescues 50% of relapsed/refractory (r/r) cHL (Venkataraman et al., 2014; Mottok and Steidl, 2018). Treatment of r/r cHL faces several obstacles as patients usually have already received substantial amounts of chemotherapy or are ineligible for brentuximab vedotin and immune checkpoint inhibition (ICPi), respectively, due to comorbidities (Armand et al., 2018; Chen et al., 2019; Kuruvilla et al., 2021). Long term toxicity (i.e. secondary neoplasia, congestive heart failure) is another so far unmet clinical complication of standard treatment (Aleman et al., 2003; Meyer et al., 2012). This calls for alternative treatment strategies with a more favorable safety and toxicity profile aiming at long-term survival.

cHL has a characteristic histology of a small minority of CD30 positive Hodgkin cells surrounded by a large number of reactive immune cells distinguishing it from most other lymphomas. This inflammatory stroma with abundantly present regulatory T-cell (Tregs), actively inhibiting the cytotoxic activity of tumor inflltrating lymphocytes (TILs) illustrates the immunologic imbalance in this disease (Cader et al., 2018; Calabretta et al., 2019). This imbalance can be addressed by novel treatment concepts for r/r cHL.

The present concept, called biomodulation or anakoinosis (from Greek ‘communication’), takes into account that heterogeneous genetic events (i.e. chromosomal aberrations) initiate characteristic tissue patterns in tumors via so-called ‘master modulators’, especially transcriptional modulators, that regulate cellular interactions on a basic level (Heudobler et al., 2018). Pro-anakoinotic therapies aim at redirecting the so called hallmarks of cancer. The biomodulatory approach tries to ‘normalize’ dysregulated, neoplasia-associated homeostatic processes via combination of regulatory active drugs, correspondingly called ‘master modifiers’ of tumor tissue (Heudobler et al., 2019b).

If combined correctly, regulatory active drugs, that show no or poor monoactivity when administered alone, may induce ^18^F-fluoro-2-deoxy-d-glucose positron emission tomography (FDG-PET) negative remissions in r/r cHL and diffuse large cell B-cell lymphoma with a very favorable toxicity profile unlike classical combination therapies in cHL (Ugocsai et al., 2016; Schelker et al., 2018).

Here we present in total six cases with r/r cHL that advance our experience on reprogramming tumor homeostasis. In the three newly treated patients, we show a continuous complete remission (cCR), achieved without any dose-intensive consolidation treatment in an elderly, severely comorbid patient, a patient with relapsed cHL after allogenic hematopoietic stem cell transplantation (allo-HSCT) and a third patient who received MEPED (metronomic chemotherapy, everolimus, pioglitazone, etoricoxib, dexamethasone) (Table 1) as a salvage therapy to become eligible for allo-HSCT. We applied a combination of low-dose metronomic chemotherapy, pioglitazone, everolimus, dexamethasone, and etoricoxib as previously published (Guarini et al., 2012; Ugocsai et al., 2016). We also provide a long-term follow-up for the three patients already published by some of the authors of this study (Ugocsai et al., 2016).

**TABLE 1:** MEPED-Regimen (28-days cycle).

Drug name	Dose	Days per cycle	Comments
Pioglitazone	45 mg	1–28	
Treosulfan	250 mg	1–28	Mild antiemetic on demand (i.e. metoclopramid)
Everolimus	15 mg	1–28	To achieve nadir levels of 15 ng/ml
Etoricoxib	60 mg	1–28	
Dexamethasone	0.5 mg	1–28	

### Treatment and Response Assessment

All six patients received MEPED administered as salvage therapy. MEPED is an all-oral treatment schedule, consisting of metronomic low dose treosulfan 250 mg p.o. daily, everolimus 15 mg p.o. daily to achieve serum trough levels of 15 ng/ml, pioglitazone 45 mg p.o. daily, etoricoxib 60 mg p.o. daily and dexamethasone 0.5 mg p.o. daily (Table 1). Medications were given continuously from day 1 in 28-days cycles. One patient with relapse after allo-HSCT received trofosfamide 50 mg daily instead of treosulfan to avoid an increased risk of myelotoxicity. All administered drugs were approved by the European Medicines Agency (EMA). Since there are multiple suppliers for all used components of MEPED a single supplier cannot be specified, as in Germany prescriptions are usually written for the active component of the medication, not a specific supplier. The purity of all drugs was according to the standards for orally administered medication in Europe.

**FIGURE 1:**
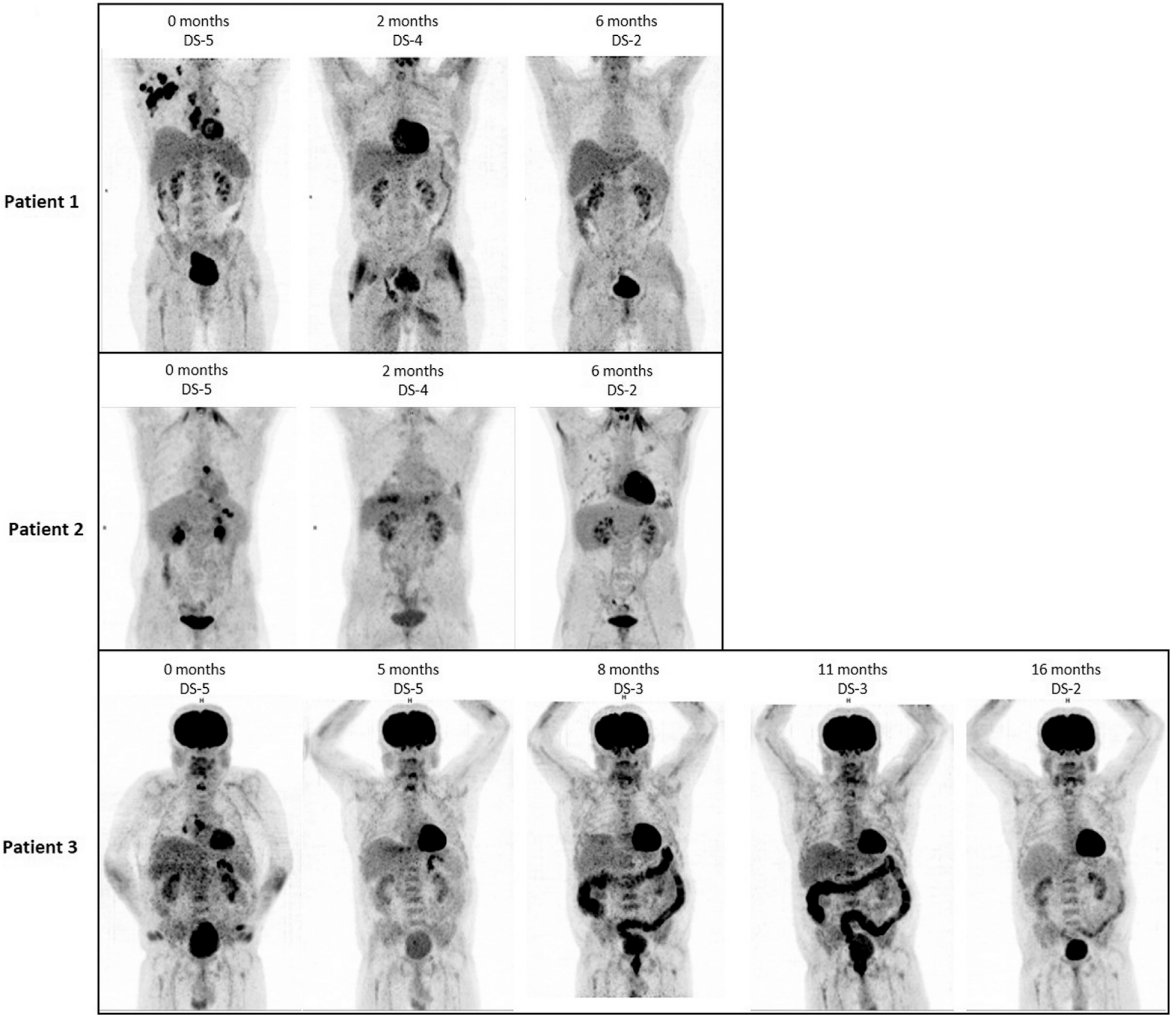
FDG PET/CT images (ventral maximum intensity projection, MIP) of patient 1–3 at the time of MEPED treatment, including Deauville Score (DS) analysis. All three patients show a decrease in DS at restaging as a sign of response. In patient 2, therapy-associated bipulmonary inflammation is found 6 months after initiation of therapy.

Tumor response was assessed by [18F] fluorodeoxyglucose–positron emission tomography (PET)/computed tomography (CT) at baseline, weeks 8–12 and in follow-up (Figure 1). Patients with PET-avid lesions at baseline were followed with PET/CT (Cheson et al., 2014). CR was defined by a negative PET Deauville score (DS) of DS-1 to 3 (Meignan et al., 2009). Data were analyzed retrospectively.

Patients were tracked weekly for adverse events and adverse events (AE) were assessed continuously during the experimental MEPED therapy up until three months after the last MEPED treatment using the National Cancer Institute Common Terminology Criteria for Adverse Events (version 4.03). Data cut-off was August 27th 2020.

### Patients’ Characteristics and Treatment Results

**FIGURE 2:**
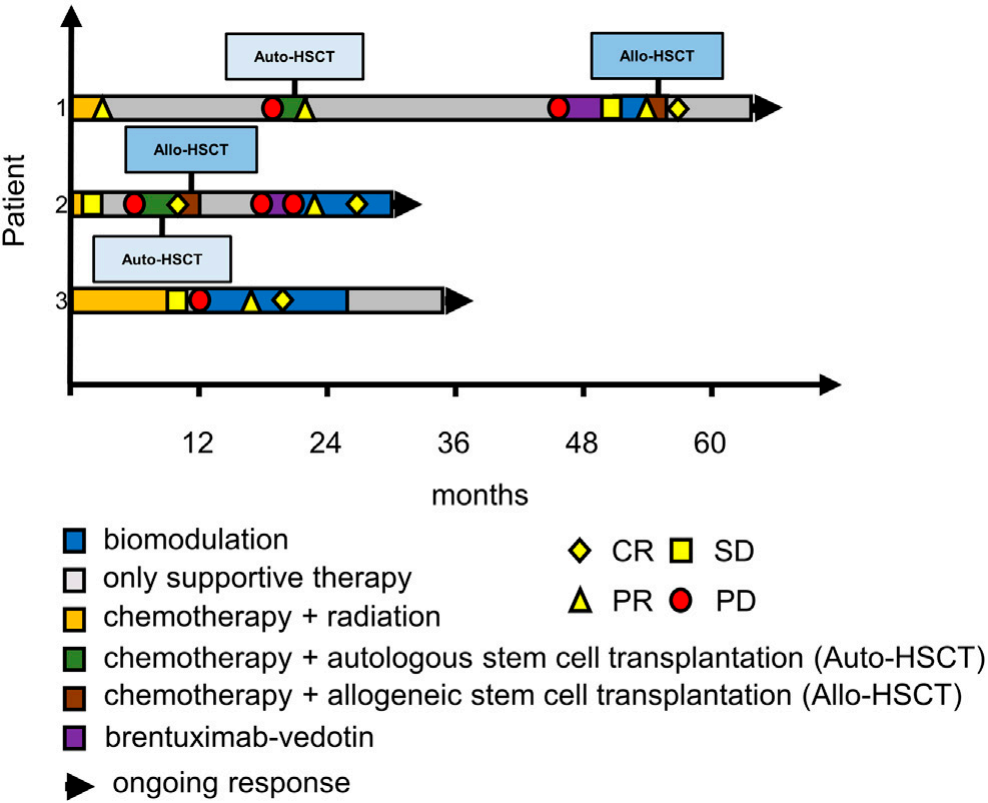
Swimmer plots of patients 1 to 3. Patient 1 had residual disease with PET DS-4 after 2 months on MEPED and achieved cCR following haploidentical allo-HSCT; patient 2 achieved cCR with MEPED following relapse after allo-HSCT, patient 3 remained in CR during consolidation treatment with MEPED and is now >12 months in CR without any Hodgkin therapy. CR: PET negativity (DS-1 to 3) plus/minus residual tumor in CT-scans. PR: PET positivity (DS-4 to 5) plus PR according to RECIST in CT scans. SD: stable disease. PD: progressive disease. DS: PET Deauville score (1–5).

Six patients at our university hospital who had refractory or relapsed Hodgkin’s disease and were not deemed eligible for standard treatment or immediate allo-HSCT by our institutional tumor board were offered MEPED therapy. No further inclusion or exclusion criteria were applied (Figure 2) (Ugocsai et al., 2016; Armand et al., 2018). Concerning the three newly treated patients, two patients were ineligible for immune-checkpoint inhibition (ICPi) due to preexisting autoimmune conditions (severe myasthenia gravis and rheumatoid arthritis) (Makarious et al., 2017; Dahi et al., 2019). The third patient had previously received an allo-HSCT. The risk for triggering severe graft-versus-host disease (GvHD) after PD-1 blockade in this patient was deemed unacceptably high by the treating physicians. The three patients published in Ugocsai et al. (2016) were also PD1-inhibitor naïve when receiving MEPED because nivolumab had not yet been approved for treating Hodgkin’s disease at the time. All patients were treated outside a clinical trial in an individual healing attempt, but with institutional review board approval. Patient characteristics are summarized in Table 2.

**TABLE 2:** Summary of patient characteristics; patients 4–6 have already been published in Ugocsai et al. (2016).

Patient No	1	2	3	4	5	6
Age at diagnosis (years)	55	21	71	27	39	37
Sex	Male	Male	Male	Male	Female	Female
Stage at initial diagnosis	IIB	IVA	IIIB	IVAE	IVAE	IVB
Stage of relapse(s)	IIIA (2)	IVB	IVB	IVA	N/A^a^	IVA
Lines of therapy before MEPED	BEACOPP/ABVD	BEACOPP	ABVD/AD	BEACOPP	BEACOPP	BEACOPP
	DHAP + auto-HSCT	DHAP + auto-HSCT		DHAP	DHAP + auto-HSCT	DHAP
	Brentuximab vedotin					Brentuximab-vedotin
Previous allo-HSCT	No	Yes	No	No	No	No
Previous autologous HSCT	Yes	Yes	No	No	Yes	No
Duration of MEPED treatment [months]	2	9	14	3	3	10
Previous ICPi	No	No	No	No	No	No

a Primary refractory disease

Patient 1 is a 55-year-old male with stage IIB cHL (nodular sclerosing, EBV negative) at initial diagnosis. He achieved partial remission (PR) after two courses of BEACOPP escalated (dose-escalated bleomycin, etoposide, doxorubicin, cyclophosphamide, vincristine, procarbazine, prednisone) and two courses of ABVD (doxorubicin, bleomycin, vinblastine, dacarbazine) followed by paraaortal and pelvic irradiation with 27 and 30 Gy, respectively. Two relapses (stage IIIA) in quick succession (16 and 43 months after completion of 1st line therapy) were treated by reinduction followed by autologous HSCT (two courses DHAP (dexamethasone, cytarabine, cisplatin) and by eight courses of brentuximab-vedotin (BV). Since BV only yielded stable disease (PET/CT), treatment was switched to MEPED. This treatment achieved partial remission (residual disease in CT and DS-4) as prerequisite for allo-HSCT. After haploidentical allo-HSCT (Gauthier et al., 2018), the patient achieved continuous CR (cCR). Response had been lasting for 8 months at data cutoff (Figure 1, upper row; Figure 2, first row).

Patient 2 is a 21-year-old male with EBV-negative stage IVA (lung involvement) cHL. His disease progressed after four courses of BEACOPP escalated followed by mediastinal and supraclavicular irradiation (30 Gy). Reinduction with 2 courses of DHAP achieved partial remission in PET/CT. Consolidation therapy by high dose chemotherapy (BEAM, carmustine, etoposide, cytarabine, melphalan) and autologous HSCT (Josting et al., 1998; Argiris et al., 2000) lead to PET negativity as a prerequisite for the haploidentical allo-HSCT thereafter. The consecutive relapse (6 months after allo-HSCT) was treated with three courses of brentuximab-vedotin. Another relapse occurred after 3 months (stage IVB). Due to prior acute intestinal GVHD PD-1 blockade was contraindicated and MEPED was initiated, resulting in PR with residual disease and DS-4 after 2 months on MEPED and PET negativity (DS-2) after 6 months of MEPED-treatment (Figure 2). The patient is currently in cCR for 5 months and is continuing MEPED therapy as a maintenance treatment (Figure 1, middle row; Figure 2, middle row). After completion of 6 months of maintenance treatment beyond achievement of CR, discontinuation of therapy is planned. By then he will have received a total of 12 months of MEPED treatment.

Patient 3 is a 71-year-old male suffering from severe myasthenia gravis and stage IIIB EBV-associated cHL at initial diagnosis. This combination decisively limited therapeutic options for specific Hodgkin therapy from the start. Only mixed response could be achieved following six cycles of ABVD and two cycles of AD. Rapid progression within two months after completion of therapy occurred showing additional pulmonary involvement (stage IVB). Since standard salvage treatments were not indicated the patient received MEPED. Five months on treatment achieved a PR with residual disease (DS-5). MEPED was continued and PET negativity was achieved 3 months later. As the patient is not eligible for autologous or allo-HSCT, he received another 8 months of adjuvant MEPED therapy. He is currently >12 months in cCR without any therapy (Figure 1, lower row; Figure 2, lower row). Additionally, his myasthenia gravis has also improved.

### Patients 4–6 From (Ugocsai et al., 2016)

**FIGURE 3:**
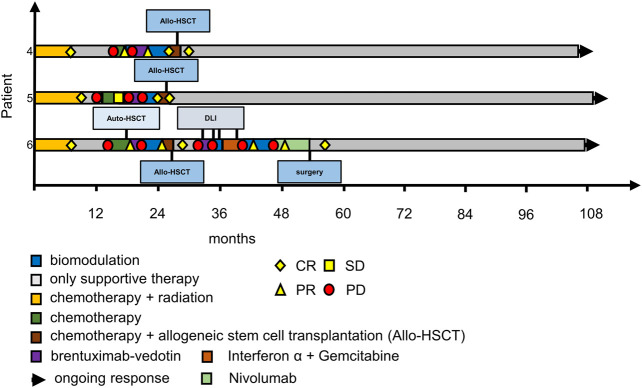
Swimmer plots of patients 4–6. Patient 4 had achieved CR on a second course of MEPED and was consolidated by allo-HSCT, he is in remission ever since; patient 5 achieved cCR with MEPED and was consolidated by allo-HSCT, she has been suffering from multiple GVHD complications since, but remains in cCR, patient 6 had relapsed several times after allo-HSCT but achieved cCR after treatment with Nivolumab and resection of residual PET positive cervical lymph nodes. CR: PET negativity (DS-1 to 3) plus/minus residual tumor in CT-scans. PR: PET positivity (DS-4 to 5) plus PR according to RECIST in CT scans. SD: stable disease. PD: progressive disease. DS: PET Deauville score (1–5); DLI: donor lymphocyte infusion; please also see Ugocsai et al. (2016).

At data cut**-**off all three patients were still in cCR. Patient No. 5 [patient No. 1 in Ugocsai et al. (2016)] has been suffering from severe chronic GvHD complications after allogeneic stem-cell transplantation, while Patient No. 4 [Patient No. 3. in Ugocsai et al. (2016)] has fully recovered without any immunosuppression. Patient No. 6 [patient No. 2 in Ugocsai et al. (2016)] relpased after allogeneic stem cell transplantation and needed multiple treatment attempts until a cCR could be achieved. Because of lack of alternatives in the end she was treated with nivolumab and one residual cervical lymph node was excised. Since then, she has achieved cCR (Figure 3). Histological work-up of this lymph node confirmed residual Hodgkin’s lymphoma at this site. This unusual strategy is noteworthy as HL is generally not treated by surgical resection. There is very limited evidence in the literature, that surgery can be used as an “ultima ratio” to treat r/r Hodgkin’s lymphoma (Fahy et al., 2019; Papadakis et al., 2021). As this patient had exhausted all systemic treatment options surgical resection of a single PET-positive site was justified.

Safety: Most grade 1 and 2 toxicities observed were hematological. Due to a sinusitis and bacterial pneumonia in patient 2 (Figure 1), trofosfamide therapy had to be interrupted for 10 days and was resumed thereafter on the original dose level. Further dose reductions were not necessary as no additional Grade 3 or 4 hematological or non-hematological toxicities occurred. MEPED treatment was administered in an out-patient setting in all three patients. No treatment related hospitalizations were necessary.

## Discussion and Conclusion

These three different cases demonstrate the versatility, clinical efficacy and low toxicity of the MEPED schedule in r/r cHL.

We show that, MEPED may induce cCR in r/r cHL without needing aggressive consolidating therapies like autologous and allo-HSCT. The necessary duration of an adjuvant MEPED therapy, however, remains a question to be answered in the future.

Secondly, MEPED may serve as salvage therapy even after allo-HSCT in cHL. This result is supported by experiences with a similar biomodulatory regimen in acute myelocytic leukemia (AML) in the same treatment line. The AML schedule also includes targeted therapy with dual transcriptional modulation, like in r/r cHL (Heudobler et al., 2019a). In AML, only two cycles were necessary to induce hematologic CR. In the present case seven four-week cycles MEPED were sufficient to achieve PET negativity. The patient simultaneously developed a chronic (GvHD) although receiving 2 immunosuppressive agents (everolimus and low dose dexamethasone). One can argue that this is a sign of an additional graft-vs.-cHL (GvL) effect. Whether MEPED triggered GvHD/GvL or this was an independent phenomenon, remains speculative.

The third case confirms efficacy of MEPED for bridging patients with r/r cHL to allogeneic transplantation (Ugocsai et al., 2016; Shah and Moskowitz, 2018). Including the recently published data on MEPED followed by allo-HSCT the pre-transplant PET negativity or massive cytoreduction may be translated in long-term overall survival of more than 4.5 years in the already published patients (Ugocsai et al., 2016) and 8 months in the present patient 2. Only one relapse occurred in the previously published population, which could be rescued by reduction of immunosuppression.

Adding these three patients to the long-term follow-up of patients in the cohort initially published by Ugoscai et al. MEPED achieves a complete response rate of 83% confirmed by PET negativity in a very negatively selected patient population.

Although approved standard treatments are highly efficacious, cumulative toxicity and treatment-associated malignancies remain a therapeutic challenge in cHL (Rueda et al., 2015; van Leeuwen and Ng, 2016; Borchmann et al., 2018; Mehta-Shah and Bartlett, 2018; Moskowitz et al., 2019; Domingo-Domènech and Sureda, 2020). Since cHL is frequently occurring in younger adults these long-term adverse effects need to be addressed for further advancing therapy. Treatment of elderly and comorbid patients with r/r cHL remains also challenging with few standard therapies available (Carter et al., 2020). The possibility of inducing on-going CR at low toxicity in comorbid patients highlights the versatility of the MEPED regimen.

Yet there are some limitations due to the retrospective data evaluation and the small case series. Further, we can only show in one refractory disease that MEPED regimen may induce cCR without any following consolidating therapy. Four patients consecutively received allo-HSCT. In these cases, MEPED was represented a very effective bridging to transpant strategy. Only four patients received brentuximab–vedotin as rescue therapy prior to MEPED, no patient nivolumab. However, MEPED induced CR after allogeneic relapse.

When looking at the costs of therapy, the MEPED regimen (ca. 2900€ per 28 days) is significantly cheaper than Nivolumab (ca. 6,500€ per 28 days) or brentuximab-vedotin (ca. 8500€ per 21 days; pat. weight 70 kg). Moreover, the application of MEPED does not require additional supportive therapy or any infusion center, thereby reducing costs again.

The suggested mechanisms of action of MEPED are, as already shown, anti-inflammatory with pioglitazone (Teresi et al., 2006; Gottfried et al., 2011) and etoricoxib (Hashemi Goradel et al., 2019), and multifaceted immunomodulatory with pioglitazone (Bahrambeigi et al., 2020; Fu et al., 2020), everolimus (Pópulo et al., 2012), dexamethasone and metronomic low-dose chemotherapy (Ge et al., 2012).

Further, the components lend themselves to combination with ICPi. It has been shown that they are able to improve T-cell function and presumably up-regulate immune-checkpoints (Peng et al., 2015; Hirayama et al., 2016; Chowdhury et al., 2018; Renner et al., 2019; Batyrova et al., 2020).

In summary, MEPED addresses important issues in cHL therapy. The biomodulatory nature of the regimen focuses on concerted activity of several biomodulatory drugs, therefore, avoiding maximal tolerable doses. MEPED reduces toxicity while keeping efficacy in r/rHL high, irrespective of line of treatment and eligibility for autologous or allogeneic HSCT.

Therefore, diversified possibilities are available for deepening knowledge about MEPED. A randomized phase II trial for r/r HL and/or the integration of MEPED in checkpoint inhibitor therapies as suggested synergistic combination partners is warranted.

## Data Availability

The data analyzed in this study is subject to the following licenses/restrictions: The datasets generated for this study are available on request to the corresponding author. Requests to access these datasets should be directed to daniel.heudobler@ukr.de.
